# Targeting reactive oxygen species (ROS) to combat the age-related loss of muscle mass and function

**DOI:** 10.1007/s10522-020-09883-x

**Published:** 2020-05-23

**Authors:** Anastasia Thoma, Tania Akter-Miah, Rebecca L. Reade, Adam P. Lightfoot

**Affiliations:** Musculoskeletal Science & Sports Medicine Research Centre, Dept. of Life Sciences, Faculty of Science & Engineering, Manchester Metropolitan University, Manchester, UK

**Keywords:** Sarcopenia, Reactive oxygen species, Oxidative stress, Mitochondria, Ageing

## Abstract

The loss of muscle mass and function with age, termed sarcopenia, is an inevitable process, which has a significant impact on quality of life. During ageing we observe a progressive loss of total muscle fibres and a reduction in cross-sectional area of the remaining fibres, resulting in a significant reduction in force output. The mechanisms which underpin sarcopenia are complex and poorly understood, ranging from inflammation, dysregulation of protein metabolism and denervation. However, there is significant evidence to demonstrate that modified ROS generation, redox dis-homeostasis and mitochondrial dysfunction may have an important role to play. Based on this, significant interest and research has interrogated potential ROS-targeted therapies, ranging from nutritional-based interventions such as vitamin E/C, polyphenols (resveratrol) and targeted pharmacological compounds, using molecules such as SS-31 and MitoQ. In this review we evaluate these approaches to target aberrant age-related ROS generation and the impact on muscle mass and function.

## Introduction

The age-related decline in muscle mass, strength and function, termed sarcopenia, is a significant contributor to frailty (Lexell 1993). Approximately 600 million people over the age of 60 years old suffering from sarcopenia in 2000 and by 2050 this population is estimated to grow to 2 billion people (Dhillon and Hasni 2017). Sarcopenia contributes considerably to the frailty seen in the elderly population, with sarcopenia being associated with generalised weakness, impaired mobility, poor balance and stamina (Walston 2012). Consequently, this can lead to an increasing number of falls, disability and mortality (Cruz-Jentoft et al. 2014).

Loss of muscle mass typically begins from around 50 years of age, occurring at a rate of around 1–2% per year, with around a 50% reduction by 80 years of age (Lexell 1993). Ageing muscle displays a reduction (atrophy) in overall fibre cross-sectional area, with a predominant loss of type II muscle fibres (Lexell 1993). Consequently, this results in an overall slow twitch phenotype, characterised by a reduction in force generation and activation velocity (Nilwik et al. 2013). The impact of sarcopenia is compounded via unloading and disuse of muscle as a consequence of a progressively sedentary lifestyle (Breen et al. 2013).

The mechanisms that underpin sarcopenia are not fully understood, however, it is a complex multifactorial network of interacting dysfunctional systems. Specifically, modified protein synthesis (Breen and Phillips 2013) and anabolic blunting (Cuthbertson et al. 2005), alongside chronic low-grade inflammation (Roubenoff 2003), mitochondrial dysfunction (Sakellariou et al. 2017), aberrant ROS generation (Vasilaki and Jackson 2013), denervation (Pollock et al. 2017; Scalabrin et al. 2019) and a reduced regenerative capacity (Almada andWagers 2016) are some of the key processes implicated in sarcopenia. In this review we will evaluate the ROS generation in skeletal muscle and the role these processes play in sarcopenia and current avenues for therapeutic intervention.

### Reactive oxygen and nitrogen species (RONS) generation and regulation in skeletal muscle

Reactive oxygen and nitrogen species are radical and non-radical bi products of cellular respiration, formed via the molecular reduction of atmospheric oxygen. Skeletal muscle as an excitable tissue with a high energetic demand is widely described as a significant generator of RONS. In the context of ageing, seminal studies in the 1950’s developed the field and investigation of RONS, formulating the “free radical theory of ageing” (Gerschmann 1954; Haraan 1955). An increased activity of reactive oxygen species (ROS) has been implicated in the processes underlying ageing and, in all species, tissues (including skeletal muscle) of aged organisms contain increased amounts of oxidative damage to lipids, DNA and proteins (Vasilaki et al. 2006).

There are a diverse family of RONS in skeletal muscle, with superoxide (O_2_.^−^) and nitric oxide (NO^−^) are the primary species generated at rest and in response to contractile activity (Sakellariou et al. 2014). Moreover, there is a complex network of enzymatic and non-enzymatic antioxidant defence mechanisms which regulate RONS generation (Halliwell and Guttridge 2015).

*Superoxide* is generated from complex I, II and III of the electron transport chain and the nicotinamide adenine dinucleotide phosphate NAD(P)H oxidases, xanthine oxidase and lipoxygenase enzymes (Hellsten et al. 1997; Zuo et al. 2004; Muller et al. 2004; Goncalves et al. 2015). Superoxide has a relatively long biological half-life of ~ 10^−6^ s, is membrane impermeable, bar movement via specific membrane channels (TOM40/VDAC/BAX). Superoxide has a low oxidising capacity towards cellular macromolecules; however, it may react with other species such as NO^−^, forming the highly reactive peroxynitrite (ONOO^−^) (Halliwell and Guttridge 2015). Typically, superoxide is dismutated to hydrogen peroxide (H_2_O_2_) by the superoxide dismutase (SOD) family of enzymes (Fukai and Ushio-Fukai 2011). The SOD family of enzymes are characterised by their ability to convert O_2_.^−^ to H_2_O_2_ are localised to different cellular compartments and differentiated by the transition metal bound to the active site. SOD1 termed CuZnSOD is localised to both the cytosol and mitochondrial intermembrane space; SOD2 termed MnSOD is enriched within the mitochondrial matrix and SOD3 (extracellular SOD) has a CuZn cofactor and is found in the interstitial spaces (Halliwell and Guttridge 2015). Approximately 35% of all cellular SOD activity is localised to the mitochondria to skeletal muscle (Ji et al. 1998). Modification of SOD activity and function has been shown to induce dysfunction in skeletal muscle. Specifically, SOD1 gene knockout mice display elevated markers of oxidative damage and an overall accelerated sarcopenic phenotype and is a model for amyotrophic lateral sclerosis (Deepa et al. 2019). In response to contractile activity, skeletal muscle upregulates the expression of both SOD1 and 2, with a higher activity in type I slow oxidative muscle fibres (Kojda and Hambrecht 2005).

*Nitric oxide* (NO^−^) is generated by the nitric oxide synthase (NOS) enzymes, catalysing the conversion of *L*-arginine to citrulline (Korhonen et al. 2005). NO^−^ has a biological half-life of ~ 1–10^−1^ s and functions via *s*-nitrosylation of cysteine residues (Stamler and Meissner 2001). There are three key members of the NOS enzyme family: inducible (iNOS), neuronal (nNOS) and endothelial (eNOS). NO^−^ has a well-established function in vasodilation and the immune response and has an important role in muscle physiology. A recent study using the nNOS^−/−^ mouse, demonstrated NO^−^ helps to regulate muscle fibre type, fatiguability and post-exercise recovery (Moon et al. 2017). NO^−^ levels are elevated in the muscle fibres of old mice following contractile activity, associated in with elevated marked of nitrosylation (3-nitrotryrosine) and eNOS activity (Pearson et al. 2015).

*Hydrogen peroxide* is a non-radical species, generated from dismutation of superoxide by the SODs, and as a bi product of protein folding in the endoplasmic reticulum via the thiol-disulphide exchange mechanism (Hudson et al. 2015). Comparative to other radical RONS species, H_2_O_2_ has a relatively long half-life 10^−5^ s and is membrane permeable. H_2_O_2_ levels are regulated by the enzymes catalase, glutathione peroxidase (Gpx) and the peroxiredoxin (PRXs) family, converting it to water and oxygen. Elevated H_2_O_2_ generation has been observed in the muscle of old mice and is reported to be derived from increased mitochondria superoxide generation (Mansouri et al. 2006). Moreover, H_2_O_2_ has been heavily implicated as an important mediator of muscle ageing, based on several studies of the transgenic mCAT model, which overexpress the human form of catalase in the mitochondria (Basisty et al. 2016). mCAT mice have been shown to have increased longevity (Schriner et al. 2005) and improved muscle function—the latter due to decreased ca^2+^ leakage from the sarcoplasmic reticulum (Umanskaya et al. 2014).

*Peroxynitrite* (ONOO^−^) is a highly reactive radical species, formed via the reaction between superoxide and nitric oxide, with a half-life of 10^−2^ s. Thiol (-SH) groups are highly sensitive to peroxynitrite (resulting in disulphide formation), which can rapidly deplete the overall thiol pools within the cell. Peroxynitrite induces the nitration of tyrosine and *S*-nitrosylation of cysteine residues and can induce significant damage to DNA and in some instances, affect enzyme activity and supress signalling cascades. Studies in isolated muscle fibres have demonstrated that peroxynitrite directly suppresses ca^2+^ stimulated force generation (Dutka et al. 2011). Studies of old mice (26–28 month) and in the SOD1^−/−^ model have shown a three-fold increase in 3-nitrotyrosine (3-NT) residues compared with young/WT controls (Sakellariou et al. 2011; Pearson et al. 2015).

*Hydroxyl radical* (OH^−^) is a highly reactive species, with a very short half-life ~ 10^−9^ s. The hydroxyl radical is formed via the fenton reaction:

$$ {\text{Fe}}^{{2 + {\text{ }}}}  + {\text{H}}_{2} {\text{O}}_{2}  \to {\text{Fe}}^{{3 + {\text{ }}}}  + {\text{OH}} \cdot  + {\text{OH}}^{{ - {\text{ }}}}   $$

Transition metals such as iron (Fe^+^) or copper (Cu^2+^) can reduce H_2_O_2_ to the hydroxyl radical. Contracting skeletal muscle has been shown to generate the hydroxyl radical (O’Neill et al. 1996) and it has been reported to modify calcium sensitivity and force generation in rodent muscle fibres (Murphy et al. 2008). However, there are some elements of controversy in terms of the fenton reaction, given the relatively low concentrations of transition metals detected in vivo (Halliwell and Guttridge 2015).

### Mitochondrial dysfunction in skeletal muscle ageing

Skeletal muscle is richly abundant in mitochondria, due to the high energetic demand of contractile activity. Thus, given the role of mitochondria in ROS generation, there has been significant interest in understanding what if any part mitochondria may play in muscle wasting and dysfunction. Aberrant ROS generation and oxidative damage have been associated with many aspects of mitochondrial dysfunction in skeletal muscle ageing. Firstly, accumulation of mitochondrial DNA mutations (mtDNA) in human aged skeletal muscle is evident (Bua et al. 2006; Herbst et al. 2007; Melov et al. 1995). Various in vitro studies have shown association with mtDNA mutations and with reduced content of the etc. complexes, oxidative phosphorylation, and membrane potential (Hiona et al. 2010; Sahu et al. 2018), as well as increased expression of mitochondrial markers of oxidative damage, decreased antioxidant defense system and mitochondrial biogenesis, indicative to a defective adaptive response to mitochondrial dysfunction in mtDNA mutator mice (Kolesar et al. 2014). These studies are consistent with results in aged rats that showed reduced levels of mitochondrial biogenesis and function markers, accompanying with swollen mitochondria (Zhu et al. 2019; Sahu et al. 2018). Furthermore, there is an age-related decline in mitofusin 2 (Mfn2) a key regulator of mitochondrial fusion (Sebastian et al. 2016), which an important process which helps regulate and accommodate the energetic demands of the cell. Moreover, ablation of Mfn2 in mice induced an ageing phenotype, associated with impaired mitophagy (Sebastian et al. 2016). The latter observation may be related the accumulation of damaged mitochondria during ageing. Changes have been observed in both intermyofibrillar (IMF) and subsarcolemmal (SS) mitochondria in aged mice. IMF mitochondria appeared longitudinal and branched in shape, whereas SS mitochondria had a larger and less circular appearance—in, contrast they did not observe changes in absolute Mfn2 levels. However, there was alteration in the ratio of genes which regulate mitochondrial fusion, fission and biogenesis—suggesting dysregulation of the overall system (Leduc-Gaudet et al. 2015). Further rodent studies have also demonstrated ageing and the loss of muscle mass and function to be associated with a downregulation of genes encoding key mitochondrial function (Ibebunjo et al. 2013).

### Targeting RONS to combat sarcopenia

Given the ever-ageing population and the socioeconomic impact of sarcopenia, there is an urgent need to develop effective targeted therapies. Moreover, the significant evidence of aberrant RONS generation and mitochondrial dysfunction in muscle ageing suggest that therapies to target these processes are worthy of investigation.

### Vitamins

Nutritional antioxidants are considered as a potential approach in an effort to combat the age-related loss of muscle mass and function, due to their antioxidant and anti-inflammatory activity. A study showed that both Vitamin E and C supplementation could improve muscle function by increasing antioxidant activity and preventing oxidative stress in aged rats (Ryan et al. 2010). A further insight into the effects of the two subgroups of vitamin E, tocopherol and tocotrienols, suggested that both α-tocopherol and tocotrienol-rich fraction (TRF) can encourage myogenic differentiation during replicative senescence, with TRF having superior effects (Khor et al. 2016). Data demonstrated that TRF can promote muscle regeneration during oxidative stress-induced premature senescence, by both increasing the proliferation capacity of myoblasts and maintaining the renewal of satellite cells (Lim et al. 2019). A further study using a vitamin E analog, TROLOX, showed that it could prevent oxidative stress and maintain skeletal muscle mass in an animal ageing model with muscle specific *Opa1* deletion (Tezze et al. 2017). A clinical trial on female athletes has shown that supplementation of Vitamin E and C together or Vitamin C alone could reduce muscle damage, alongside with decreased levels of oxidative stress markers, induced by aerobic exercise (Taghiyar et al. 2013). Further enhancing previous evidence of beneficial effects of vitamins supplementation in muscle ageing, a recent study showed a positive association between high dietary intake of vitamin E, C, and carotenoids with improved sarcopenic indices (muscle mass and power); however, this association was only noticeable in women younger than 65 years (Welch et al. 2019).

However, it is important to note that there is compelling evidence demonstrating the use of broad-spectrum antioxidants such as vitamin C and E, can in fact suppress the beneficial adaptations in response to exercise training (Ristow et al. 2009). In contrast, mitoQ supplementation during endurance training, displayed no tangible beneficial or negative effects in terms of adaptations (Shill et al. 2016). Moreover *n*-acetylecysteine supplementation has been reported to suppress fatigue following repeated bouts of intermittent exercise (Cobley et al. 2011). However, RONS are no longer considered to be purely toxic/damaging molecules and instead are important mediators and regulators of an array of signalling cascades and pathways (Cobley et al. 2015).

### Resveratrol

Resveratol has been widely studying in muscle ageing due to its antioxidant activity, as well as its effects on peroxisome proliferator-activated receptor gamma coactivator-1a, an important regulator of mitochondrial biogenesis (Gülçin 2010). The anti-ageing effects of resveratrol supplementation for 4 weeks, have been recently reported, highlighting its capability of inhibiting lipid peroxidation and increasing catalase and superoxide dismutase activity, by targeting mitochondrial mass and function, associated with improved physical endurance in aged mice (Muhammad and Allam 2018). In an in vitro study, resveratrol has shown dose-dependent effects on muscle cell plasticity, with low doses preventing ROS generation and inducing muscle regeneration, effects not observed in high doses (Bosutti and Degens 2015). However, various studies have obtained controversial results on the effect of resveratrol on ageing-associated muscle dysfunction. A 6–7-week low-to-moderate daily resveratrol intake has produced no effects in muscular strength and function in aged mice (Baumann et al. 2014; Zhou et al. 2019). Similarly, long-term (10 months) low-to-moderate daily resveratrol intake, even though it could mitigate ageing-induced oxidative stress, no beneficial effects were observed on muscle mass and function in aged mice, while mitochondrial biogenesis was also unaffected by resveratrol (Park et al. 2018). Overall, it seems that resveratrol has protective effects against ageing-induced abnormalities, even the incongruent findings, as they can likely be explained by variations in the age of experimental species, as well as the dosage and duration of supplementation; those variations highlight the need for further investigation for acute and chronic resveratrol intake both in vitro and in vivo with consistent experimental approach.

### Targeted antioxidant therapies

When evaluating studies of antioxidants, there is a large degree of heterogeneity in terms of efficacy and outcomes. This is likely due in part to a poor distribution and uptake of such compounds into cells and tissues, resulting in the need to administer such antioxidants in high concentrations. In the case of the latter, this may be a reason for the observed pro-oxidant effect of some purported antioxidants (Pearson et al. 2006). Thus, development of targeted antioxidant therapies has provided a novel and promising avenue of research, in an effort to combat the pitfalls of broad-spectrum approaches.

Given the aforementioned potential role of mitochondrial dysfunction in sarcopenia, the use of novel antioxidant compounds to this organelle have been investigated in sarcopenia and a range of myopathies and neuromuscular disease (Sakellariou et al. 2017). The Szeto-Schiller (SS-) peptide family are small molecules, with potent antioxidant capacity towards a range of radical species (Lightfoot et al. 2015). The Dmt-D-Arg-Phe-atnDAP-NH_2_ structure of the SS- peptides results in their accumulation at the inner mitochondrial membrane (IMM). The accumulation of SS- peptides at the IMM does not rely on the mitochondrial membrane potential, which offers a unique opportunity to target mitochondria which may have underlying damage (Zhao et al. 2004).

SS-31 has been explored as a potential therapy against the age-related loss of muscle mass and function (sarcopenia). Modified ROS generation and oxidative damage has been widely characterised as an important mediator of muscle wasting and dysfunction (Lightfoot et al. 2014). In this study, mice were administered sub-cutaneous SS-31 peptide (1.5 mg/kg), over a four-month period from 24 to 28 months of age. Data showed significant reductions in markers of oxidative damage in the muscles of old mice, however, this did prevent the age-related loss of muscle mass and function (Sakellariou et al. 2016a). This clearly supports the potent ability of SS peptides as antioxidants, however, the lack of change in underlying pathology in this instance may be due to the complex and multi-factorial nature of sarcopenia. In contrast, 8-weeks of SS-31 (3 mg/kg) treatment in 26-month old mice, showed improvements in mitochondrial structure, function and homeostasisalongside an increased in exercise tolerance (Campbell et al. 2019). Moreover, short-term (1-h) SS-31 treatment (3 mg/kg) of 27-month-old mice, improved mitochondrial function and muscle performance (Siegel et al. 2013). Thus, differences in dose and duration of treatment with SS-31 in these models may be significant factors in the observed differences in outcome.

More recently, SS-31 has been explored in the context of a murine model of doxorubicin-induced toxicity (Montalvo et al. 2019). Doxorubicin is a highly potent chemotherapy agent, which has significant side-effects on muscle. In this study, SS-31 (3 mg/kg) attenuated mitochondrial ROS generation and proteolytic pathway activity in the muscles of doxorubicin treated mice (Montalvo et al. 2019). Furthermore, studies of leukocytes taken from patients with type 2 diabetes, demonstrated a reduction in inflammation, via down regulation of TNF-alpha and NF-kB and upregulation of SIRT1, in response to treatment with SS-31 peptide (Escribano-Lopez et al. 2018). Thus, there are clear and compelling evidence demonstrating the potent antioxidant ability of SS family of peptides in a range of disorders, which can have direct functional effects.

Mitoquinone is a compound derived from ubiquinone, which has a lipophilic cation (triphenylphosphonium) conjugated to it, which drives accumulation of the molecule within mitochondria (Murphy and Smith 2007). Ubiquione is a crucial component of the electron transport chain as a two-electron carrier, as well as having antioxidant capacity (Nicholls and Ferguson 2002). MitoQ readily accumulates within the cytosol of the cell driven by the plasma membrane potential, however, is found in > 100-fold greater concentration within the mitochondria at the matrix face of the inner membrane, driven by the mitochondrial membrane potential (Murphy and Smith 2007). Upon accumulation in the mitochondria, MitoQ is converted to its active antioxidant form (ubiquinol) via reduction at complex II (Murphy and Smith 2007). When acting as an antioxidant, MitoQ is oxidised to ubiquinone and then rapidly reduced back to ubiquinol, rejuvenating its antioxidant capacity and thus, is a marker of the efficacy of MitoQ. MitoQ has an affinity for an targets the hydroxyl (.OH) radical, thus preventing lipid peroxidation (Tauskela 2007), alongside quenching superoxide generation (James et al. 2005). In the context of ageing-induced mitochondrial dysfunction, a recent study has clearly found that MitoQ was able to alleviate ageing-induced changes in respiratory chain, ATP production, and membrane potential, highlighting the role of peroxynitrite in ageing deficits (Maiti et al. 2018). However, its effects on ageing-induced muscle atrophy seem to be less clear. A study of ageing mice (24–26 months) reported failure of long-term MitoQ supplementation to protect against age-related loss of muscle mass function and oxidative damage (Sakellariou et al. 2016b). A further novel mitochondria-targeted antioxidant is XJB-5-131, which is TEMPO derivative that has a gramicidin S moiety conjugated to it, which encourages accumulation in the mitochondria (Robinson et al. 2018). XJB-5-131 has been found to decrease mitochondrial ROS and membrane depolarization (Escobales et al. 2014), increase the activity of the electron transport chain complexes, and improve the single fibre contractile properties in aged rats (Javadov et al. 2015).

## Conclusions

**Fig. 1:**
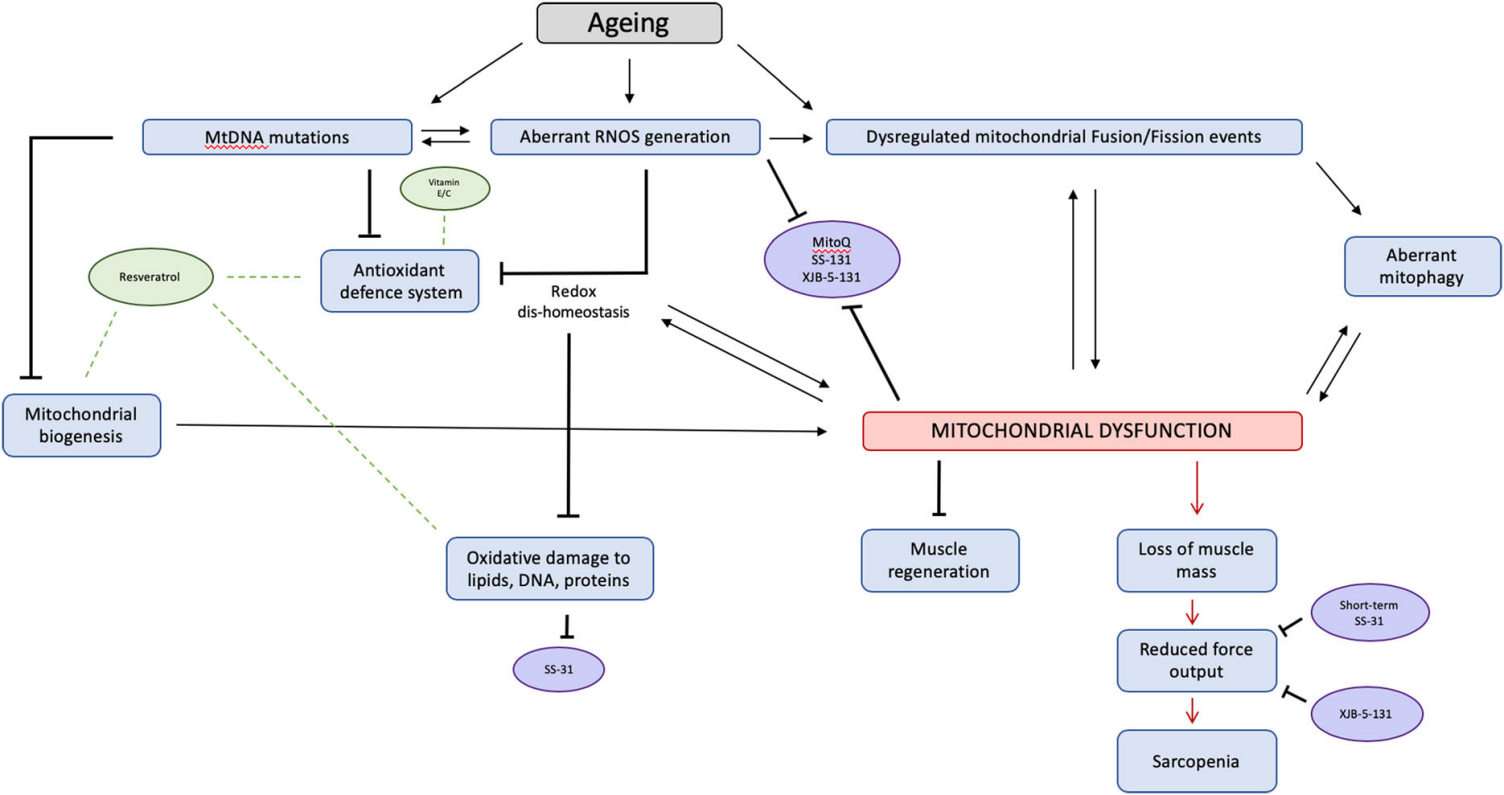
A schematic summary of the role of ROS and putative interventions in the age-related loss of muscle mass and function

Aberrant ROS generation and redox dis-homeostasis have clear and important role to play in the age-related loss of muscle mass and function (Fig. 1). However, there is no clear consensus on intervention to target ROS. There remains significant discordance in findings, likely due to differences in model systems (species) in tandem with dosing regimens. Moreover, this raises questions on the ability to translate these mechanistic findings to humans—where there is a need to pursue this line of research further.
